# Demographic and Clinical Determinants of Conjugated Pneumococcal Vaccine Uptake and Short-Term All-Cause Mortality in Vaccinated and Unvaccinated Cohorts in Patients with Heart Failure and Reduced Ejection Fraction: A Prospective Cohort Study

**DOI:** 10.3390/medicina61050869

**Published:** 2025-05-09

**Authors:** Yalçın Velibey, Erkan Kahraman, Melih Oz, Murat Gokalp, Kader Ozturk, Muhsin Melik, Utku Ulukoksal, Ufuk Egemen Yazar, Furkan Fatih Yucedag, Elif Ozoguz, Emre Ozguclu, Mutlu Seyda Ocalmaz, Mehmet Eren, Osman Bolca, Tolga Sinan Güvenç

**Affiliations:** 1 Department of Cardiology, Siyami Ersek Thoracic and Cardiovascular Surgery Center, Training and Research Hospital, Istanbul 34668, Türkiye; erkan.kahraman@hotmail.com (E.K.); melih_oz3334@hotmail.com (M.O.); drmuratgokalp@gmail.com (M.G.); dr.kaderozturk2@gmail.com (K.O.); drmuhsinmelik@gmail.com (M.M.); utkuulukoksal@gmail.com (U.U.); ufuk789yazar@gmail.com (U.E.Y.); fatihycdg@hotmail.com (F.F.Y.); drelfozoguz@gmail.com (E.O.); emre_ozguclu@hotmail.com (E.O.); meseren@hotmail.com (M.E.); drbolca@gmail.com (O.B.); 2 Department of Infectious Diseases and Microbiology, Siyami Ersek Thoracic and Cardiovascular Surgery Center, Training and Research Hospital, Istanbul 34668, Türkiye; 3 Department of Cardiology, Faculty of Medicine, Istinye University, Istanbul 34396, Türkiye; tsguvenc@gmail.com

**Keywords:** heart failure, pneumonia, pneumococcus, vaccination

## Abstract

*Background and Objectives*: Patients with heart failure (HF) are at risk of increased morbidity and mortality related to pneumococcal pneumonia, and routine vaccination with a conjugated pneumococcal vaccine (PCV) for HF patients is strongly endorsed by all major international guidelines. Despite this, data on the factors associated with vaccine uptake remain scarce. The aim of this study was to understand the demographic and clinical factors associated with vaccine uptake in patients with HF and analyze the all-cause mortality in the vaccinated and unvaccinated cohorts. *Materials and Methods*: Four hundred and fifty patients with HF and a reduced ejection fraction followed up at a single center were enrolled. Patients were followed up for a median of 164.0 (148.0–181.0) days. *Results*: In total, 193 of the 450 patients (42.9%) were vaccinated with PCV-13 at enrollment. Vaccinated patients were more likely to have an implantable device, namely an implantable cardioverter/defibrillator (ICD), cardiac resynchronization treatment (CRT) or left ventricular assist device (LVAD), and less likely to have a past medical history of hypertension and chronic obstructive pulmonary disease (COPD) at baseline. After multivariable adjustment, the presence of an ICD (OR: 3.17, 95% CI: 1.98–5.08), CRT (OR: 2.75, 95% CI: 1.45–5.20) and COPD (OR: 0.42, 95% CI: 0.19–0.94) remained as determinants of vaccination. All-cause mortality was not different across vaccinated or unvaccinated patients either in the unmatched (log-rank *p* = 0.67) or matched (log-rank *p* = 0.52) cohorts. *Conclusions*: The presence of implantable devices and coexisting COPD was associated with a higher and lower likelihood of vaccination with PCV-13, respectively. No difference in mortality across cohorts was observed in this observational analysis.

## 1. Introduction

Heart failure (HF) is a major health concern, with an estimated 64 million patients experiencing HF worldwide. Even in recent studies, the 5-year mortality rate of HF patients remains at 43% despite remarkable advances in medical–surgical management [1,2,3]. While two-thirds of patients with HF will eventually die due to pump failure or sudden cardiac death; infections, particularly pneumonia, are the immediate cause of death in more than 10% of patients [4,5]. Respiratory infections are preventable in theory as effective vaccines are available for the immunization of influenza, pneumococcal pneumonia and COVID-19 [6]. In the Organized Program to Initiate Lifesaving Treatment in Hospitalized Patients with Heart Failure (OPTIMIZE-HF) study, pneumonia accounted for 15.3% of hospitalizations in HF and was associated with a 60% increase in in-hospital mortality [7]. In addition to its immediate effects, pneumonia leads to oxidative stress and immune-mediated injury, which cause endothelial dysfunction, thrombogenesis and the destabilization of atherosclerotic plaques, which may lead to the worsening of HF via the aggravation of ischemia [8,9]. The effectiveness of conjugated pneumococcal vaccines (PCVs) ranges between 41% and 71%, and available evidence suggests a significant decrease in mortality with PCV-13 in patients at a high risk of cardiovascular events, including patients with HF [10,11,12]. Due to the simplicity of the intervention involved and the proven benefits of vaccination, all major guidelines highly recommend vaccination against pneumococcal pneumonia in HF patients [13,14]. Despite this, a significant fraction of patients with HF refuse vaccination, and little is known about the factors associated with vaccine uptake in HF patients. In the present study, we analyzed patients with chronic HF and reduced ejection fraction (EF) to determine the factors associated with PCV-13 vaccine uptake. As a secondary aim, we sought to understand the association between vaccination and short-term all-cause mortality in HF.

## 2. Materials and Methods

### 2.1. Patient Selection

For the present study, patients with heart failure were prospectively enrolled at a large academic cardiology center between 15 March and 31 July 2024 and followed up until the 30 October 2024. Patients over 18 years old, with a documented EF ≤ 40% on transthoracic echocardiography and symptoms consistent with HF were included. Patients with a life expectancy of less than 6 months and those with an uncertain vaccination status were excluded. As PCV-13 has only been commercially available for the last 5 years, patients who had at least one shot of PCV-13 were accepted as vaccinated. Patients’ demographic and clinical data were collected and recorded by direct interviews or by using the institutional electronic medical database. This study was conducted according to the Declaration of Helsinki and its subsequent revisions, and all participants gave their written consent prior to enrollment. An ethics committee approved the design and conduct of the study.

### 2.2. Definitions

HF with reduced EF was defined as the presence of symptoms and/or signs of HF for at least 3 months before enrollment, with objective echocardiographic evidence showing an EF ≤ 40%. A repeat screening echocardiography was performed prior to enrollment to confirm that the patient had not experienced EF recovery at the time of inclusion. The guideline-directed medical treatment (GDMT) score was calculated by summing the number of treatments known to reduce mortality in patients with HF and a reduced ejection fraction. All-cause mortality was defined as mortality due to any cause other than traumatic deaths. All participants and/or their relatives were contacted at the end of the study to determine survival and the cause of death if the participant was deceased. If the participants and/or their relatives could not be contacted, the electronic database provided by the Directorate General of Civil Registration and Citizenship Affairs was used.

### 2.3. Statistical Analysis

Continuous variables were given as mean ± SD and categorical variables were presented as percentages. For conventional analyses, patients were grouped into two groups according to their vaccination status. Student’s *t*-test was used to compare continuous variables between groups, while chi-squared or Fisher’s exact tests were used for categorical variables. A multivariable logistic regression model was built to determine the independent predictors of PCV-13 vaccination. Variables that trended towards significance (i.e., with a *p*-value < 0.10) in the univariable analyses were included in the multivariable model, and variables that had a significant association with vaccination were considered independent predictors. In addition to this multivariable model, a second analysis based on discriminant analysis was performed to build a model that would be able to predict PCV-13 vaccination among HF patients with the highest accuracy, and the results of this analysis were expressed as a confusion matrix. All clinically relevant parameters were initially included in this latter model, and a stepwise selection using Wilks’ lambda was used to determine the important parameters related to vaccination. Kaplan–Meier curves and life tables were drawn to determine the association between PCV-13 vaccination and all-cause mortality in the overall cohort and a second propensity-matched cohort. The propensity score was calculated using 12 variables with a matching tolerance of 0.1, and priority was given to exact matches. In addition, exploratory analyses for three specific subgroups (patients over 65 years, patients with COPD and patients that remain active smokers) were performed to understand the effectiveness of PCV-13 in these subgroups. All statistical analyses were performed using Jamovi (The jamovi project (2024). jamovi. (Version 2.5) for MacOs. Retrieved from https://www.jamovi.org, accessed on 8 June 2024) and SPSS 27.1 for MacOs (IBM Inc, Armonk, NY, USA) software.

## 3. Results

**Figure 1:**
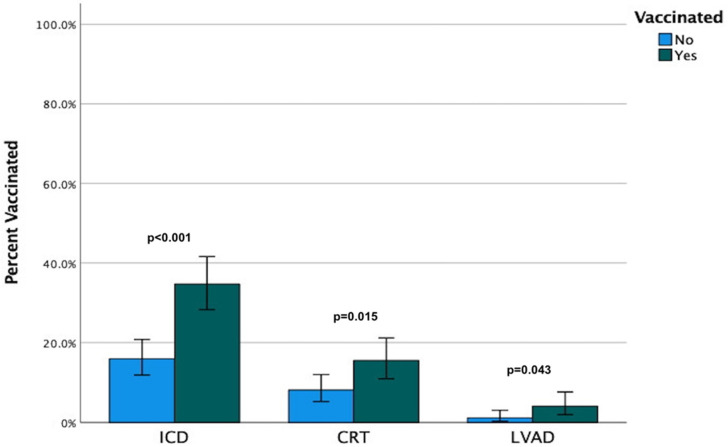
Frequencies of vaccination with a 13-valent conjugated pneumococcal vaccine in heart failure patients with a reduced ejection fraction who had an implantable cardioverter/defibrillator, cardiac resynchronization device, or left ventricular assist device implanted. Error bars represent 95% confidence intervals.

**Figure 2:**
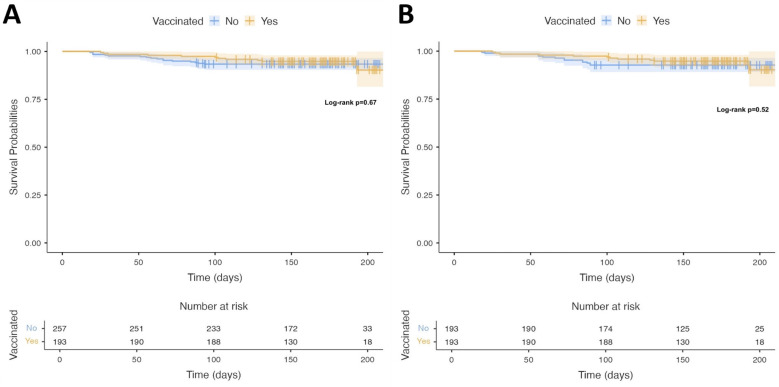
Kaplan–Meier curves showing survival in patients who did or did not receive the 13-valent conjugated pneumococcal vaccine in the overall cohort (**A**) and in the propensity-score-matched cohort (**B**). Numbers below the curves show numbers at risk for each time period.

In total, 193 of the 450 patients (42.9%) were vaccinated for PCV-13. The baseline demographic and clinical characteristics of the study population are summarized in Table 1. Patients who were vaccinated had a lower EF and were more likely to have a cardiac implanted electronic device, namely an implantable cardioverter/defibrillator (ICD), cardiac resynchronization device with defibrillator (CRT-D) or left ventricular assist device (Figure 1). Interestingly, they were also less likely to have a history of hypertension or chronic obstructive pulmonary disease (COPD). In the univariable analysis, the factors associated with an increased likelihood of vaccination were the past implantation of a pacemaker capable of cardiac resynchronization therapy (CRT) or an implantable cardiac defibrillator (ICD), the presence of a left ventricular assist device, or a lower EF, while those with a past medical history of hypertension or COPD were less likely to be vaccinated. The independent predictors of vaccination were the presence of a cardiac implantable electronic device and a past medical history of COPD (Table 2). On discriminant analysis, a model that consisted of the presence of either an ICD or CRT, hypertension, COPD, or chronic renal disease correctly classified 66.9% of patients according to their vaccination status, with a Nagelkerke pseudo-r^2^ of 0.138 and a model Wilks’ lambda of 0.896 (Table 3). Vaccination was not associated with all-cause mortality either in the overall study sample (log-rank *p* = 0.67) (Figure 2A) or in the propensity-score-matched cohort (log-rank *p* = 0.52) (Figure 2B). In three exploratory subgroup analyses, vaccination with PCV-13 was not associated with all-cause mortality in patients over 65 years (log-rank *p* = 0.89), patients with COPD (log-rank *p* = 0.58), or patients that were active smokers (log-rank *p* = 0.23).

## 4. Discussion

In the present study, we investigated the factors associated with PCV-13 vaccine uptake and short-term all-cause mortality in HF patients with reduced EF. The main findings of the present analysis are as follows: (i) the implantation of a permanent device, whether an implantable pacemaker/defibrillator, cardiac resynchronization device, or ventricular assist device, is associated with increased vaccine uptake; (ii) the presence of several comorbidities, such as a past medical history of hypertension or chronic obstructive pulmonary disease, was associated with a lower likelihood of vaccination, (iii) demographic and clinical factors can explain only a small subset of the variability (i.e., 14%) in vaccine uptake; and (iv) pneumococcal vaccination was not associated with short-term all-cause mortality in the present study sample, although various limitations of the present study limit the robustness of this latter finding. See the Graphical Abstract for a summary of the main findings.

Vaccine uptake for a given population is associated with various individual, psychosocial and cultural factors, and cannot be explained solely with demographic or clinical determinants [15]. However, demographics and clinical factors affect the perceived risk to an individual, with a heightened perceived risk being an important determinant of increased vaccine uptake [16,17]. In a retrospective analysis of the Prospective Comparison of ARNI with ACEI to Determine Impact on Global Mortality and Morbidity in Heart Failure (PARADIGM-HF) trial, several demographic and clinical factors, including age, race, a worse functional status, the presence of diabetes, and the presence of an implantable cardioverter defibrillator, were found to be predictors of vaccine uptake [18]. Likewise, a retrospective analysis of a nationwide Danish population study found that various comorbidities (including hypertension and diabetes) and implantable devices were more common in HF patients who received the influenza vaccination. However, this latter study did not provide a separate analysis of the demographic/clinical determinants of vaccine uptake [19]. In contrast, prior to this study, there were no comparable data on the factors associated with pneumococcal vaccine uptake.

The present findings suggest that patients with implantable devices were more likely to be vaccinated, while specific comorbidities were linked to a reduced vaccine uptake. In contrast to most other cardiac and non-cardiac procedures, implantable cardiac electronic devices and left ventricular assist devices can be “seen” or “felt” (in the case of pacemaker-based devices) by the patient and may serve as a constant reminder of their underlying HF, which may enhance the perceived risk associated with HF. As mentioned, a similar finding was also noted for influenza vaccinations in HF patients in at least two studies [18,19]. In contrast, it remains uncertain why patients with several comorbidities (such as hypertension or COPD) but not others (chronic renal disease) were less likely to be vaccinated with PCV-13. Given that virtually all patients with a reduced ejection fraction would not have an elevated systolic blood pressure at the time of enrollment, regardless of a past diagnosis, a causal association between these two conditions (i.e., an altered risk perception due to hypertension and vaccination) is very unlikely. In fact, a history of hypertension does not appear to independently predict vaccine uptake, although it remains useful as a component of the model. It is more difficult to rule out a direct causal relationship between COPD and lower PCV-13 vaccine uptake because these patients were already aware that they had a second respiratory disease, which could alter their perceived risk of infection. It is also likely that factors not measured in the present study (such as education or socioeconomic status) could be responsible for these associations, given that social factors are determinants of vaccine hesitancy [19,20,21]. Finally, random associations could not be ruled out, emphasizing the need for further studies on the factors associated with vaccine uptake.

It should also be emphasized that regardless of the presence or absence of causal associations between the aforementioned factors and PCV-13 vaccination, only a small amount of variation in vaccine uptake could be explained solely by demographic or clinical factors. Sociocultural factors, such as individuals’ level of education or health literacy, influence vaccine uptake in the general population, as lower levels of education are associated with a fear and mistrust of vaccines [22,23]. In addition, issues related to clinician bias towards age and gender subgroups or difficulties in accessing healthcare are other potentially important social determinants that may influence vaccine uptake [24,25]. Although comparable data in HF patients are not widely available, it is reasonable to assume that these factors may explain some of the remaining variation in vaccine uptake. The lack of data on these social and cultural determinants may explain why the present model, consisting only of demographics and clinical parameters, had a rather modest association with PCV-13 vaccine uptake, and as such, further studies incorporating social and cultural determinants are needed to better understand the factors related to pneumococcal vaccination.

Although there is widespread observational evidence demonstrating an association between influenza vaccination and reduced mortality in HF patients, the only randomized controlled trial failed to show a reduction in mortality in HF patients with influenza vaccination [26]. The available data for pneumococcal vaccination are more limited, but at least two retrospective studies in patients have suggested that vaccination with a pneumococcal vaccine (either polysaccharide or conjugated) lowers mortality [12,27]. Randomized clinical trials specific to pneumococcal vaccination in HF patients are not available. However, one trial that included high-risk patients >65 years old (including HF patients) could not demonstrate a reduction in death or serious adverse events with PCV-13 [28]. Although we were unable to demonstrate a difference in mortality in HF patients who were vaccinated with PCV-13, the present results should be interpreted with caution due the observational nature of the present analysis, the small sample size, and the relatively short (median 165 days for survivors) follow-up duration. All these latter factors limit the power of the present study to show a meaningful difference between the two groups. Finally, sources of bias not directly related to the statistical power of the study, such as a decrease in respiratory infections during the spring or summer seasons when the overall temperature is warmer, could explain this lack of difference in mortality. Nevertheless, the present results emphasize the need for adequately powered studies and randomized trials with long-term follow-up data that could provide high-quality evidence of the benefit of pneumococcal vaccination in HF patients.

From a practical perspective, the present findings could be used to improve vaccination rates in patients with HF. Patients with implanted devices appear to be receptive to vaccination with PCV-13, so frequent reminders could easily improve vaccination rates in this subgroup. It is also reasonable to assume that these patients would be more receptive to vaccination against other respiratory pathogens. In contrast, COPD patients with HF, who are among those most likely to be harmed by vaccine hesitancy, appear to be less likely to be vaccinated with PCV-13. Pneumococcus is the major cause of severe pneumonia in COPD patients, and PCV-13 is highly effective in preventing pneumonia in these patients [29,30]. It would be reasonable to implement healthcare-wide policy changes that provide additional education to HF patients with coexisting COPD to reduce the risk of morbidity and mortality related to pneumococcal pneumonia, as previous studies have shown that emphasizing the possible consequences of a given disease is an effective way to increase vaccination rates [31]. It should be noted that messaging designed to refute claims about vaccine complications tends to backfire by increasing patients’ distrust of vaccines [32].

## 5. Study Limitations

The present study has several limitations. This study was conducted at a single center with a limited number of patients, and the follow-up duration was relatively short. Given that the primary aim of the present study was to understand the clinical factors associated with PCV-13 vaccination in HF patients, the study was not powered enough to demonstrate a long-term difference in mortality between the groups. Also, data on other important outcome measures, such as HF-related hospitalizations, were not available. Only patients with a reduced ejection fraction were included in the present study, so the results are not necessarily generalizable to all patients with HF. The patients’ vaccination status with either influenza or polysaccharide pneumococcal vaccines was not known, which might have affected the present results (particularly those regarding outcomes) given that co-vaccination with either vaccine should increase the effectiveness of PCV-13 for preventing respiratory infections. Data concerning socioeconomic, cultural or economic variables were not collected or analyzed, so the variation in PCV-13 vaccination may be—to some extent—related to such factors. Finally, as with all observational studies, the present work could not determine the direction of the association, and correlation should not be interpreted as causality. As an example, patients with implanted devices would be expected to have more frequent contact with healthcare providers, which may explain the higher rate of PCV-13 vaccinations in this patient group—rather than the proposed “receptiveness” of these patients for vaccination.

## 6. Conclusions

Although pneumococcal vaccination is strongly recommended for HF patients in virtually all major guidelines, the vaccine uptake rate remains a concern, as multiple factors may affect patients’ decision to proceed with vaccination. The present findings suggest that patients who underwent device implantation—particularly “visible” devices such as ICDs or LVADs—were more likely to get vaccinated with PCV-13, while several comorbidities could reduce the odds of vaccination. However, the overall predictive utility of a model composed of demographic and clinical factors was modest at best, and much of the remaining variability should be attributed to other determinants not included in this study, such as sociocultural variables. While a decrease in mortality with PCV-13 vaccination could not be demonstrated in the present study, this finding reflects the limitations of the present analysis. This emphasizes the need to conduct adequately powered clinical studies and trials to fill this evidence gap.

## Figures and Tables

**Table 1:** Baseline demographics, clinical parameters, and prior medications in patients that were vaccinated or not vaccinated with 13-valent pneumococcal conjugate vaccine (PCV-13).

Parameter	No PCV-13 Vaccination (*n* = 257)	PCV-13 Vaccination (*n* = 193)	*p* Value
Demographic and clinical variables			
Age, years	58.2 ± 13.0	58.3 ± 13.3	0.751
Male gender, *n* (%)	206 (80.2)	155 (80.3)	0.967
Ischemic etiology, *n* (%)	150 (58.4)	108 (56.0)	0.609
Left ventricular ejection fraction (%)	28.1 ± 7.3	26.5 ± 7.3	0.017
History of diabetes mellitus, *n* (%)	91 (35.4)	78 (40.4)	0.278
History of hypertension, *n* (%)	152 (59.1)	94 (48.7)	0.028
Smoking, *n* (%)	48 (18.7)	31 (16.1)	0.471
Chronic obstructive pulmonary disease, *n* (%)	29 (11.3)	9 (4.7)	0.012
History of stroke, *n* (%)	17 (6.6)	10 (5.2)	0.526
On hemodialysis, *n* (%)	11 (4.3)	6 (3.1)	0.516
Prior CABG, *n* (%)	49 (19.1)	33 (17.1)	0.593
Prior PCI, *n* (%)	111 (43.2)	83 (43.0)	0.969
Presence of an ICD, *n* (%)	41 (16.0)	67 (34.7)	<0.001
Presence of a CRT, *n* (%)	21 (8.2)	30 (15.5)	0.015
Presence of an LVAD, *n* (%)	3 (1.2)	8 (4.1)	0.043
Atrial fibrillation, *n* (%)	61 (23.7)	48 (24.9)	0.781
eGFR, mL/min/1.73 m^2^	70.9 ± 27.2	66.4 ± 25.6	0.092
Prior medications *n* (%)			
ACE inhibitors, *n* (%)	134 (52.1)	71 (36.8)	0.001
ARBs, *n* (%)	31 (12.1)	23 (11.9)	0.963
ARNI, *n* (%)	50 (19.5)	63 (32.6)	0.001
Beta-blockers, *n* (%)	242 (94.2)	185 (95.9)	0.420
MRAs, *n* (%)	193 (75.1)	147 (76.2)	0.794
SGLT2 inhibitors, *n* (%)	119 (46.3)	97 (50.3)	0.406
Diuretic, *n* (%)	200 (77.8)	152 (78.8)	0.812
Ivabradine, *n* (%)	31 (12.1)	22 (11.4)	0.829
Digoxin, *n* (%)	22 (8.6)	30 (15.5)	0.022

Abbreviations: PCV-13—13-valent pneumococcal conjugate vaccine; CABG—coronary artery bypass graft; PCI—percutaneous coronary intervention; ICD—implantable cardioverter-defibrillator CRT—cardiac resynchronization therapy; LVAD—left ventricular assist device; eGFR—estimated glomerular filtration rate; ACE—angiotensin-converting enzyme; ARBs—angiotensin receptor blockers; ARNI—angiotensin receptor–neprilysin inhibitor; MRAs—mineralocorticoid receptor antagonists; SGLT2—sodium-glucose co-transporter 2.

**Table 2:** Univariable and multivariable associations between vaccination with 13-valent conjugated pneumococcal vaccine and relevant demographic and clinical parameters.

Characteristic	Univariable Analysis		Multivariable Analysis	
	Odds Ratio (95% CI)	*p* Value	Odds Ratio (95% CI)	*p* Value
Age (per years)	1.00 (0.98–1.02)	0.92		
Gender (being female)	0.99 (0.62–1.58)	0.97		
Diabetes mellitus (presence of)	1.24 (0.84–1.82)	0.28		
Hypertension (past history of)	0.66 (0.45–0.96)	0.03	0.78 (0.52–1.16)	0.22
COPD (presence of)	0.39 (0.18–0.84)	0.02	0.42 (0.19–0.94)	0.04
Atrial fibrillation (presence of)	1.06 (0.69–1.64)	0.78		
Chronic renal disease (presence of)	1.30 (0.88–1.94)	0.19		
Active smoking (presence of)	0.83 (0.51–1.37)	0.47		
Ischemic cardiomyopathy (presence of)	0.95 (0.65–1.38)	0.77		
Left ventricular EF (per unit increase)	0.97 (0.95–0.99)	0.02	0.99 (0.96–1.02)	0.58
GDMT score (each point increase)	1.05 (0.87–1.27)	0.63		
ICD (presence of)	2.80 (1.79–4.38)	<0.001	3.17 (1.98–5.08)	<0.001
CRT (presence of)	2.07 (1.14–3.74)	<0.001	2.75 (1.45–5.20)	0.002
Hemodialysis (being on)	0.72 (0.28–1.97)	0.52		
LVAD (presence of)	3.66 (0.96–13.99)	0.06	3.09 (0.76–12.58)	0.11

Abbreviations: COPD—chronic obstructive pulmonary disease; EF—ejection fraction; GDMT—guideline-directed medical treatment score; ICD—implantable cardioverter–defibrillator; CRT—cardiac resynchronization device; LVAD—left ventricular assist device.

**Table 3:** Confusion matrix for the variables included in the final step of the discriminant analysis.

Characteristic	Significance of F to Remove	Wilks’ Lambda
ICD (presence of)	<0.001	0.956
CRT (presence of)	<0.001	0.923
COPD (presence of)	0.03	0.905
CKD (presence of)	0.006	0.911
Hypertension (presence of)	0.03	0.905

Abbreviations: ICD—implantable cardioverter-defibrillator; CRT—cardiac resynchronization treatment; COPD—chronic obstructive pulmonary disease; CKD—chronic kidney disease.

## Data Availability

The data presented in this study are available upon request from the corresponding author.
